# Comparison of mass spectrometry and fourier transform infrared spectroscopy of plasma samples in identification of patients with fracture-related infections

**DOI:** 10.1371/journal.pone.0330743

**Published:** 2025-09-22

**Authors:** Sarah Malek, Roman M. Natoli, Bartek Rajwa

**Affiliations:** 1 Department of Veterinary Clinical Sciences, College of Veterinary Medicine, Purdue University, West Lafayette, Indiana, United States of America; 2 Indiana Center for Musculoskeletal Health (ICMH), Indiana University School of Medicine, Indianapolis, Indiana, United States of America; 3 Department of Orthopedic Surgery, Indiana University School of Medicine, Indianapolis, Indiana, United States of America; 4 Bindley Bioscience Center, Purdue University, West Lafayette, Indiana, United States of America; Universitas Muhammadiyah Aceh, INDONESIA

## Abstract

**Objectives:**

Fracture-related infections (FRIs) have significant impact on patient outcomes. Diagnosing FRIs is challenging due to lack of robust, minimally invasive diagnostic tests in the early stages of the disease. The objective of this study was to evaluate the ability of proteomic mass spectrometry (MS) (quantitative approach) and spectral pattern analysis based on fourier transform infrared (FTIR) spectroscopy of plasma samples (qualitative approach) in discriminating between FRI and controls.

**Materials and methods:**

A prospective case-control study at a level 1 trauma center was conducted. Patients meeting confirmatory FRI criteria were matched with controls without infection based on age, time after surgery, and fracture region. Plasma samples were collected at the time of presentation for FRI and saved for batch analysis. Tandem mass tag liquid chromatography-mass spectrometry was used for proteomics, and FTIR spectroscopy of dried films was used to obtain mid-infrared spectra from samples. Mid-infrared spectra were preprocessed, and for MS data, protein abundance ratios of FRI and controls were compared. Multivariate analysis-based predictive models were developed separately for FTIR-based spectra and MS-based protein ratio data.

**Results:**

Thirteen FRI and 13 controls were included in the study. The predictive models based on FTIR spectroscopy data had an average area under the receiver operating characteristic (AUROC) of ≈0.803, CI_95_(0.8, 0.81), the average sensitivity was ≈ 0. 0.755, CI_95_(0.75, 0.76), and the specificity was ≈ 0.677, CI_95_(0.672, 0.682). The MS-based predictive models from protein abundance ratio results had an average AUROC of ≈0.735, CI_95_(0.732, 0.737), the average sensitivity was ≈ 0.74, CI_95_ (0.739, 0.747), and the specificity was ≈ 0.653, CI_95_(0.649, 0.656).

**Discussion and conclusions:**

Mass spectrometry and spectral pattern recognition based on FTIR spectroscopy can both be used to develop predictive models that can discriminate between FRI and control samples. There is potential for both analytical approaches as candidate diagnostic biomarkers in FRI patients that require further validation in future studies.

## Introduction

**Fig 1:**
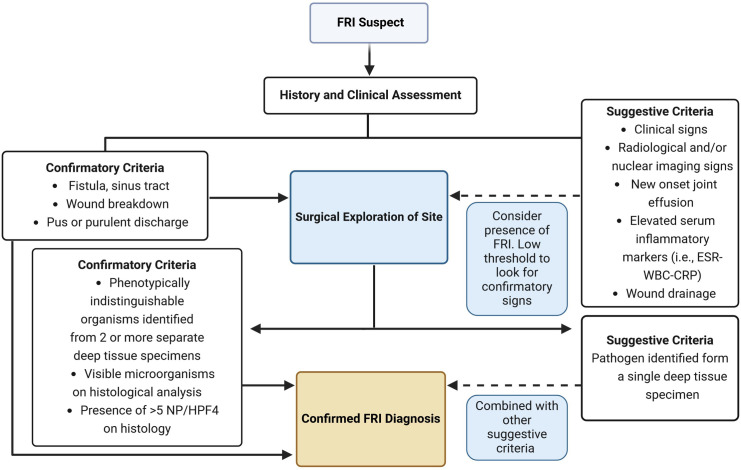
Diagnostic algorithm for a patient suspected of fracture-related infection (FRI). The algorithm is based on the updated definition of FRI [36] ESR (erythrocyte sedimentation rate), WBC (white blood cell count), CRP (C-reactive protein).

The incidence of postoperative fracture-related infections (FRIs) varies from 5–10% [1]. Depending on the site of fracture, type of infection, and the health care system involved, there is a 1.2 to 6-fold increase in costs associated with treating patients with FRIs worldwide [2–5]. Diagnosis of FRI remains a challenge due to the historically ill-defined definition of postoperative surgical site infections after fracture repair surgeries, limited investigation of contributing risk factors, and limitations of utilized diagnostic tests [6,7]. A standardized definition of FRI has only been recently defined and updated [7,8]. Based on this more widely utilized definition, there are highly specific diagnostic tests for the presence of infection (i.e., confirmatory criteria). However, in this definition, there are also findings that are suggestive of infection in the absence of confirmatory criteria (i.e., suggestive criteria). A diagram of the diagnostic algorithm for suspected FRI cases based on this FRI definition is presented in Fig 1. Patients with suggestive criteria require further investigation that typically involves more invasive procedures (i.e., multiple deep tissue biopsies for culture and histopathology), which leads to delays in definitive treatment. Considering approximately 25% of patients with suggestive criteria are eventually confirmed as FRI [9,10], it is important to improve the sensitivity and specificity of diagnostic tests to facilitate more reliable and timely diagnosis of such cases.

Blood-based biomarkers that have been utilized as suggestive criteria include white blood cell (WBC) count, erythrocyte sedimentation rate (ESR), C-reactive protein (CRP). Recent studies have shown that these three biomarkers are not sufficiently accurate predictors of FRI [11–14]. Measurement of other inflammatory biomarkers via enzyme-linked immunosorbent assay (ELISA) methodology has been utilized to evaluate other potential candidate biomarkers in FRI patients [15–18]. However, in one study that evaluated 49 proteins, only platelet-derived growth factor AB/BB (PDGF-AB/BB), and Monokine induced by gamma interferon (MIG) (in addition to CRP) showed promising results as potential novel candidate diagnostic biomarkers in predicting FRIs [15–18]. These novel candidate biomarkers still require validation in larger clinical studies. Proteomic analysis of blood samples overcomes the limitation of ELISA-based approaches by simultaneous quantitative measurement of thousands of proteins that can help facilitate the discovery of candidate biomarkers between disease and control samples. A recent study evaluating plasma samples from patients with confirmatory FRI criteria to controls demonstrated systemic activation of the complement and coagulation cascades with significant differences in abundance ratio in 32 out of more than 1000 measured proteins [19]. This approach has been utilized in medical conditions such as sepsis and trauma patients to identify prognostic biomarkers and assess response to treatment [20,21]. Another utility of proteomics-based approaches in medical research is the ability to evaluate the disease pattern and use multivariate analytical approaches to develop predictive algorithms based on qualitative or quantitative measurements [22]. Fourier-transform infrared (FTIR) spectroscopy of dried films of blood samples is an example of a qualitative pattern recognition approach to disease [23,24]. Mid-infrared (MIR) absorption of biological samples using FTIR spectroscopy produces unique patterns that are reflective of the sum of all mid-infrared (MIR)-active molecular bonds in a sample [24,25]. The FTIR spectroscopy of biological fluids (e.g., blood) is a simple, cost-effective methodology that is a clinically accessible tool previously used in diagnosing a variety of disease processes in both animals and humans [26–35]. The unique spectrum of the sample can then be used as a “fingerprint” since the disease state can alter the molecular composition of biological fluids [34]. A study comparing ELISA-based measurement of proteins versus mid-infrared spectral patterns of plasma samples using FTIR spectroscopy found that both approaches could be used to develop predictive models that performed well in distinguishing between FRI and control samples [18]. The purpose of this study was to compare the performance of mass spectrometry to FTIR spectroscopy in distinguishing between FRI and control plasma samples based on predictive models.

## Materials and methods

### Patients

This diagnostic, level III [37] study was performed at a single level-one trauma center over a nine months period, from June 25,2019 to March 24, 2020. Inclusion and exclusion criteria (Table 1) were the same for both the confirmed FRI and control groups. The confirmed FRI group had an additional inclusion criterion of a clinically suspected and subsequently confirmed FRI. No patient in this study had rheumatologic disease or other known chronic inflammatory conditions. We did not exclude patients who had received antibiotic treatment leading up to their FRI diagnosis. All FRI confirmed patients were enrolled prior to surgical intervention for their infection. ESR, CRP, and WBC, as well as three intraoperative cultures and gram stains, were obtained as part of the standard of care for the FRI patients. Patients in the control cohort were identified and matched to the FRI patients based on age (±15 years), time after surgery (±2 weeks), and fracture region. Fracture regions were matched as follows: upper extremity long bones (humerus, radius/ulna, clavicle), lower extremity long bones (femur and tibia), and other lower extremity bones (e.g., patella, ankle, tarsal bones). Control patients were identified through screening clinic schedules for patients undergoing routine fracture care follow-up. All controls had to be infection-free for a minimum of six months after enrollment as determined by routine clinic follow-up, chart review, or phone calls. Written and signed informed consent was obtained from all participating patients prior to enrollment (IRB#1905884760).

**Table 1:** Inclusion and exclusion criteria.

Inclusion	Exclusion
Age 18–85 years inclusive	Hand or Spine fracture
Extremity, pelvic ring, or acetabulum fracture that was surgically treated with retained orthopedic implant within two years of blood sample collection	Pregnancy
	Incarceration
	Known immunosuppressive state
	Ongoing treatment with immunomodulatory drug
	Localized or systemic infection
	Second or more debridement for infection
	Hemodialysis
	Venous thromboembolism
	Definitive treatment with arthroplasty, K-wires, or external fixation

### Blood collection and processing

Blood samples were obtained from the FRI cohort preoperatively on the day of surgical intervention to address the infection. Blood samples were obtained from the control cohort during routine fracture care follow-up visits. Specifically, approximately 5 ml of peripheral venous blood was collected in an EDTA purple top tube (BD Vacutainer®, Becton, Dickinson and Company, Franklin Lakes, NJ). The tube was inverted 4–5 times to allow the blood to mix with the anticoagulant before it was centrifuged at 1500 g for 10 minutes. Plasma was then extracted, aliquoted into 500 µL tubes, and stored at −80°C until batch analysis.

### Mass spectrometry

Tandem mass tag liquid chromatography-mass spectrometry (TMT LC-MS/MS) was performed by the Center for Proteome Analysis (CPA) at Indiana University School of Medicine. Protocols are described in detail in our previously published work [19] and are also available in the S1 File.

### FTIR spectroscopy

Samples were thawed in room temperature (22°C) and then diluted with potassium thiocyanate (KSCN, SigmaUltra, Sigma-Aldrich Inc, St Louis, MO), as an internal control, in a 2:1 ratio. Using a previously described technique, three 8 μL replicates of each sample were applied on a 96-welled silicon microplate (Bruker Scientific, LLC, Billerica, MA, USA) and allowed to dry at room temperature (20–22°C) for a minimum of two hours before acquiring the spectra [38,39]. Each microplate was placed in the multi-sampler (HTS-XT, Bruker Scientific, LLC, Billerica, MA, USA) attachment of an FTIR spectrometer (INVENIO S, Bruker Scientific, LLC, Billerica, MA, USA). The spectra acquisition was performed within 24 hours of the samples being loaded on each microplate. The MIR absorbance spectra in the wavenumber range of 400–4,000 cm^–1^ were recorded using the OPUS software (version 6.5, Bruker Optics, GmbH, Ettlingen, Germany). For each sample evaluation, 512 interferograms were signal averaged and Fourier transformed to produce a nominal resolution of 4 cm^-1^ for the resulting spectrum [38,40–42]. The background spectrum was measured once per plate based on a single empty well in the same location on every plate.

### FTIR data preprocessing

Chemometric analysis of the FTIR data was conducted to categorize samples based on their relationship with the health status of the subjects in the study. Furthermore, the analysis aimed to identify the characteristic features of the samples that define their composition and ultimately determine their classification outcome. It is important to note that the features may not be exploratory if transformed through the described process pipeline.

The raw data underwent preprocessing steps, which included normalization to the area under the curve, followed by normalization to the KSCN peak using the additive log-ratio method. To remove the baseline drift and noise from the data, the Savitzky-Golay [43] filter was applied, and the KSCN peak was subsequently removed. Further, the discrete cosine transform (DCT) was utilized, which converted the data into the frequency domain [44].

The DCT is a mathematical transformation that can convert a finite sequence of data points into a combination of cosine functions with varying frequencies. It is primarily used in image and signal processing applications, where it is vital for tasks such as image compression and feature extraction. Therefore, the spectral data was represented as follows:


$$X_{k}=\sqrt{\frac{\text{2}}{N}}\cdot C_{k}\sum_{n=\text{0}}^{N-\text{1}}\;x_{n}\cdot \text{co}\text{s}\left(\frac{\pi }{N}\left(n+\frac{\text{1}}{\text{2}}\right)k\right)\text{for}k=\text{0,1,}\ldots \text{,}N-\text{1}$$

where $X_{k}$ represents the transformed coefficient at frequency k, and $x_{n}$ is the original data sequence. The parameter N denotes the length of the data sequence.

The term $\sqrt{\frac{2}{N}}$ is a normalization factor that ensures the transformation is orthonormal, ensuring that the energy of the original signal x is preserved in the transformed sequence X. The term $C_{k}$ represents another coefficient that depends on the value of k. For k=0, $C_{0}=\frac{1}{\sqrt{2}}$, and for k>0, $C_{k}=1$. A frequency filtering step was then implemented to remove the low-frequency component, which exhibited near-zero variance.

Subsequently, the data underwent univariate feature filtering. This was done by analyzing the entries in the transformed spectral vector one by one using t-tests and Cohen’s *d* calculation to determine their association with the health class. Only the features that demonstrated a separation of more than *d* > 0.5 were retained for further analysis. The DCT operations were performed using R-language for statistical computing and the library DTT (Discrete Trigonometric Transforms).

### Statistical analysis

#### Classification.

The processed DC-transformed spectral vectors were used to train the elastic-net logistic regression model, which had an upper limit of 50 for the number of used coefficients. This upper limit was chosen arbitrarily, but the choice was informed by the diminishing, informative predictive content of the features. The model was optimized to minimize the objective function L defined as:


$$L\left(\beta \right)=\frac{\text{1}}{N}\sum_{i=\text{1}}^{N}\;\left[y_{i}\text{lo}\text{g}\left(p\left(y_{i}=\text{1}\right)\right)+\left(\text{1}-y_{i}\right)\text{lo}\text{g}\left(\text{1}-p\left(y_{i}=\text{1}\right)\right)\right]+\lambda_{\text{1}}\left[\frac{\text{1}}{\text{2}}(\text{1}-\alpha )\sum_{j=\text{1}}^{D}\;\left|\beta_{j}\right|+\frac{\text{1}}{\text{2}}\alpha \sum_{j=\text{1}}^{D}\;\beta_{j}^{\text{2}}\right]$$

where β is the vector of coefficients $\left(\beta_{0},\beta_{1},\ldots ,\beta_{D}\right)$, $p\left(y_{i}=1\right)$ is the predicted probability of the positive class, and λ is the regularization parameter controlling the strength of the elastic net regularization. The parameter α controls the mix between ℓ_1_ and ℓ_2_ regularization, with α=1 representing LASSO (ℓ_1_) and α=0 representing Ridge (ℓ_2_) regularization. By incorporating the ℓ_1_ and ℓ_2_ terms, the approach can achieve feature selection while performing its primary classification function. ℓ_1_ term facilitates sparsity by inducing many coefficients to become precisely zero. As a result, ℓ1 regularization tends to drive the coefficients of irrelevant or less important features to zero, effectively removing them from the model. The ℓ2 regularization method does not reduce coefficients to exactly zero; rather, it penalizes larger coefficient values. This approach is beneficial in preventing overfitting since it reduces the coefficients’ magnitude, making the model less susceptible to individual data points and noise. Although the ℓ2 term does not directly facilitate feature selection, it stabilizes the model and improves its generalization performance by lessening the influence of less relevant features.

Given the limited number of samples, we opted for bootstrap-based estimation of the classifier’s performance rather than the more commonly used k-fold cross-validation method [45]. To accommodate the small number of examples, the training utilized 100 × bootstrap sampling. We evaluated the model’s performance by calculating the mean area under the receiver operating characteristic curve (AUROC) and its standard deviation (SD). Each training session was repeated independently repeated 100 × , varying the random seeds that determined the bootstrap splits. The final classification result was reported through a meta-analysis of all individual training sessions, assuming a fixed effect model.

To visualize the classification capability of the selected frequencies, a linear discriminant function was trained with the frequencies selected by the embedded feature selection of the elastic-net regressor. This provided insight into the potential of resolving the two classes using a linear model. The classification performance of the system was expressed as AUROC, Sensitivity, and Specificity with respective 95% confidence intervals (CI_95_). Although the classification is performed using elastic net regression, we visualized the results by using the subset of selected variables, compressing the information with SVD/PCA, and employing the first 15 principal components (explaining ~99% of the variance) to build a linear discriminant coordinate space [46] (See Fig 4).

**Fig 2:**
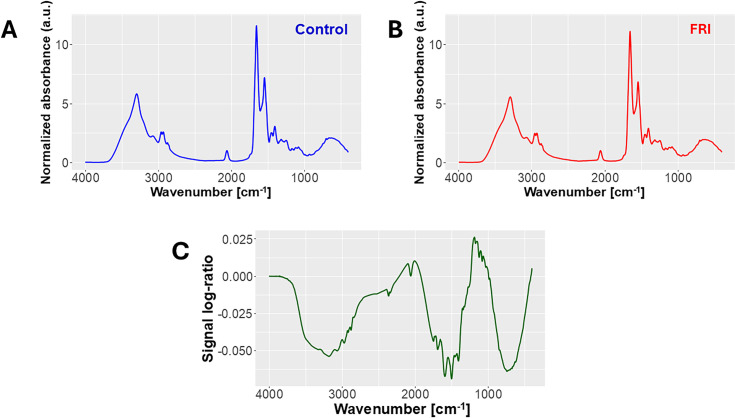
FTIR spectra visualization. Normalized averaged FTIR spectra of the control samples (**A**) and the FRI samples (**B**), along with the log ratio of the averaged spectra (**C**), highlighting differences in the FTIR signal. Arbitrary units (a.u.).

**Fig 3:**
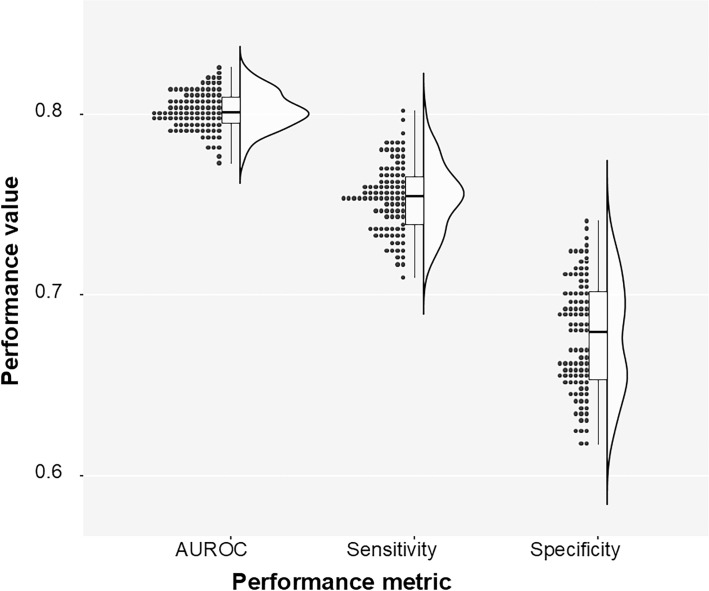
Predictive model performance for FTIR spectra. Bootstrap estimation of AUROC, sensitivity and specificity of the elastic net classifier trained on the DC-transformed data.

The analysis pipeline for the mass spectrometry (MS) data differed from that of the MIR data in that the Savitsky-Golay filtering and DCT with subsequent frequency-domain filtering step were not utilized due to the lack of formation of continuous, highly correlated spectra by the MS data. However, the remaining steps of the analysis pipeline were consistent with those employed in the MIR data analysis. Specifically, an elastic net system with embedded feature selection was employed to train the discrimination system and generate a list of the most predictive proteins in a multivariate setting. These proteins were used to perform the overrepresentation analysis employing the Reactome and STRING platforms [47,48]. The classification and related data processing operations were performed using the caret and glmnet packages in R for statistical analysis computing.

#### Evaluation of associations between FTIR and MS data.

In order to identify any simple associations between the MS and the MIR readouts, we attempted to use canonical correlation analysis (CCA) and a cross-modal autoencoder (NNE) [49,50].

CCA finds linear projections of the MIR matrix **X** and the MS matrix **Y** that are maximally correlated:


$$\rho =\underset{𝐚,𝐛}{\text{max}}\;\frac{{𝐚}^{⊤}\Sigma_{XY}𝐛}{\sqrt{\left({𝐚}^{⊤}\Sigma_{XX}𝐚\right)\left({𝐛}^{⊤}\Sigma_{YY}𝐛\right)}},$$

where $\Sigma_{XX}$, $\Sigma_{YY}$ are the within-set covariances and $\Sigma_{XY}$ the cross-covariance. We applied CCA to the most discriminatory MS proteins and raw FTIR wavenumbers (no DCT) to avoid altering the original spectral features.

A cross-modal autoencoder was trained to learn non-linear links between the two modalities. Each input (MIR or MS data) passed through three dense layers and a 2-node bottleneck; the decoder mirrored this path to reconstruct the opposite modality. The network, implemented in TensorFlow/Keras, minimized joint reconstruction error, forcing the bottleneck to capture any shared structure beyond CCA’s linear scope.

## Results

Eighty-two patients were screened for enrollment, of which 22 confirmed FRIs and 16 controls had samples obtained. Matching, as described above, resulted in 13 pairs. Eight of 13 had at least two positive cultures with phenotypically indistinguishable pathogens obtained during surgery for their infection; all 13 of the FRIs met confirmatory criteria with either fistula/sinus/wound breakdown and/or purulent drainage on initial presentation. Table 2 summarizes patient demographic, clinical, and co-morbidity data for both groups. There were no statistically significant differences in age or fracture region. There was a statistically significant difference in time-points (mean of one week) during the post-operative period at which samples were obtained (*P* = 0.045). Seven patients in the FRI group had received antibiotics within two weeks of their blood draw.

**Table 2:** Description of cohort.

Demographics	Overall Cohort (n = 26)	FRI (n = 13)	Control (n = 13)	*P* value^a^
Age	51.3 (14.9)	51.4 (14.8)	51.2 (15.5)	0.953
Sex				0.226
Male	16	10	6	
Female	10	3	7	
BMI	30.2 (7.9)	28 (4.6)	32.4 (9.9)	0.209
Weeks Post Operation	6.0 (4.3)	6.4 (4.5)	5.5 (4.4)	0.043
Clinical				
Bone Involvement				>0.999
Femur/Tibia	18	9	9	
Patella/Ankle/Foot	6	3	3	
Upper Extremity/Clavicle	2	1	1	
Implant Used				0.047
IMN	12	9	3	
Plate	14	4	10	
Fracture Type				0.096
Open	4	4	0	
Closed	22	9	13	
NSAID/Steroid Use				>0.999
Yes	1	1	0	
No	25	12	13	
Co-morbidities				
Diabetes Mellitus				0.322
Yes	5	1	4	
No	21	12	9	
History of MRSA				0.48
Yes	2	2	0	
No	24	11	13	
Tobacco Use				>0.999
Yes	7	4	3	
No	19	9	10	
Alcohol Abuse				0.48
Yes	2	2	0	
No	24	11	13	

Values are means (standard deviation) for continuous data (i.e., age, BMI, weeks post-operation). All other values are counts. BMI (body mass index), IMN (intramedullary nail), NSAID (non-steroidal anti-inflammatory drug), MRSA (multi-drug-resistant Staphylococcus aureus).

^a^Results from two-sided matched *t*-test for continuous data and Fisher’s Exact test for categorical data. Statistical significance is set at *P* < 0.05.

### FTIR spectroscopy

The collected FTIR spectra for the FRI and control groups are presented in Fig 2A and 2B, where no clear differences are immediately observable. However, computing the log ratio of the spectra for the two groups reveals distinct variations, as shown in Fig 2C.

**Fig 4:**
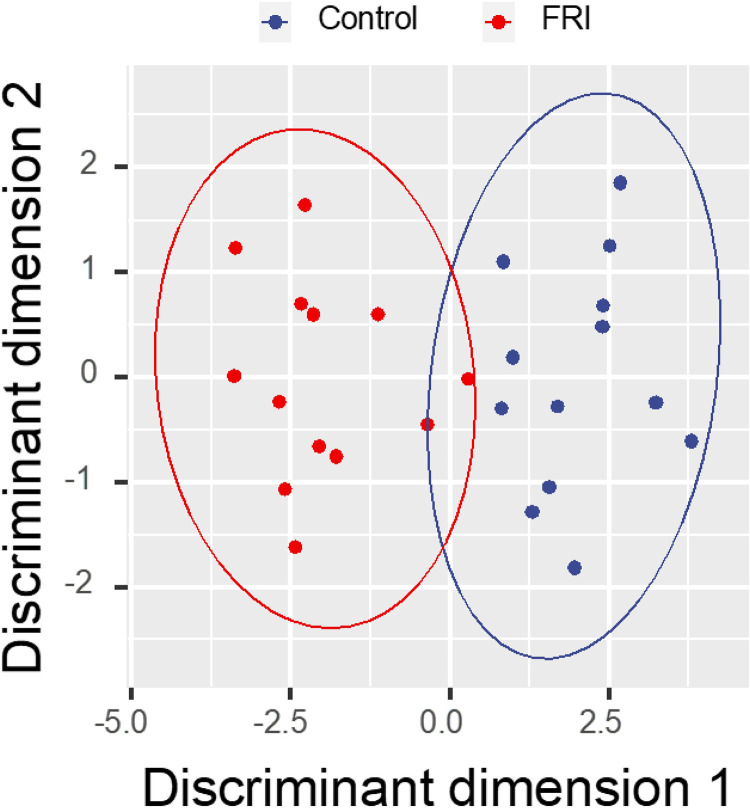
Separation of the control and FRI samples by FTIR spectra. Projection of the transformed, feature-filtered, and SVD-compressed FTIR spectra onto two discriminant coordinates illustrated the separation between control and FRI and control samples. The ellipses represent the 95-percentile confidence boundaries for the data groups, calculated based on a multivariate t-distribution.

The meta-analysis of the multiple bootstrap runs showed that the average AUROC was ≈ 0.803, CI_95_(0.8, 0.81), the average sensitivity was ≈ 0. 0.755, CI_95_(0.75, 0.76), and the specificity was ≈ 0.677, CI_95_(0.672, 0.682). The classification performance of the system is illustrated in Figs 3 and 4.

### Mass spectrometry

**Fig 5:**
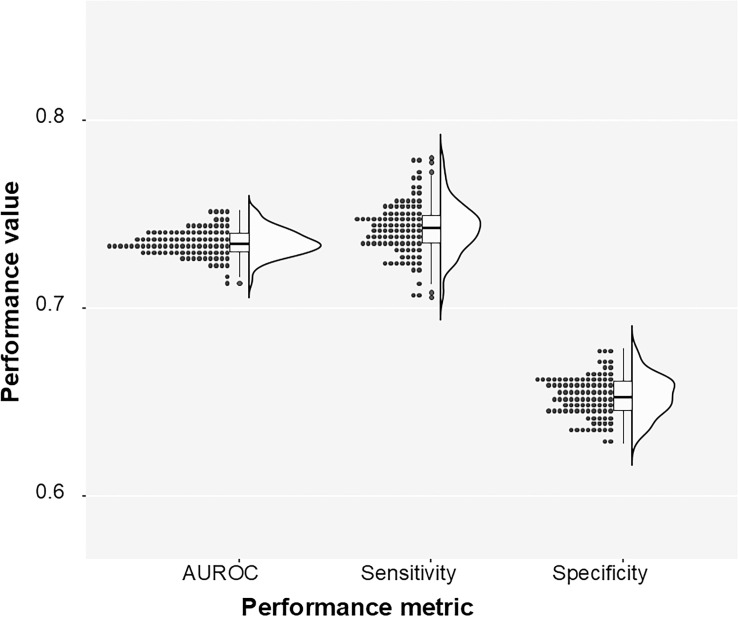
Predictive model performance for mass spectrometry (MS) data. Bootstrap estimation of AUROC, sensitivity, and specificity of the elastic net classifier trained on the MS data.

The MS input underwent a data processing procedure comparable to the one previously described. The meta-analysis of the multiple bootstrap runs of the MS-based model showed that the average AUROC was ≈ 0.735, CI_95_(0.732, 0.737), the average sensitivity was ≈ 0.74, CI_95_ (0.739, 0.747), and the specificity was ≈ 0.653, CI_95_(0.649, 0.656). The MS-derived model classification performance is illustrated in Fig 5.

The top 40 features selected by the multiple runs of the sparse model included the proteins listed in S1 Table. These proteins were used to perform the overrepresentation analysis; the detailed results are shown in S1 Table. The pathways identified for these proteins are shown in S2 Table.

Both analyses, the linear CCA and the non-linear cross-modal auto-encoder, failed to reveal a straightforward link between the MIR spectra and the MS proteome. The canonical correlations never exceeded permutation-based significance thresholds, and although the auto-encoder achieved a low reconstruction loss, its two-node bottleneck did not separate FRI samples from controls.

## Discussion

The predictive potential of FTIR spectral data is well-established; however, its limited interpretability in the context of proteomics findings presents a considerable challenge. This issue primarily stems from the inherent complexity of our samples’ spectra, which prevents the unique attribution of individual spectral peaks to specific proteins or relevant biomarkers. Furthermore, to enhance the informational content and improve the robustness of predictive models, the spectral data undergoes additional compression through the application of the DCT. While this transformation optimizes the data for classification purposes, it further diminishes its interpretability relative to the original spectral input. It is crucial to underscore that the

FTIR spectral features identified through the elastic net approach do not correspond to readily interpretable wavelengths, and currently, no straightforward method exists to restore such interpretability.

The elastic net classifier’s embedded feature selection capability also effectively worked with MS data input and yielded a selection of proteins that jointly contributed to the distinguishability of the two experimental cohorts. Nevertheless, it is important to acknowledge that the overall accuracy achieved through proteomics was surprisingly lower than that of the MIR spectra obtained from FTIR spectroscopy (80% versus 73%, respectively). Based on this observation, two conclusions can be drawn. Firstly, it should be noted that diagnostic methods that are explainable, such as proteomics, may not always exhibit the best level of performance. Secondly, the results provide significant motivation to research further phenotypic biophysical diagnostic techniques, such as MIR spectra, which already surpass certain complex “omic” analyses due to their high prediction and cost-effectiveness.

The discrepancy between the predictive models of FTIR spectroscopy and MS can be further explained by the differences in what is detected on each platform. Proteomic analysis using MS can only detect proteins, and even then, it has limitations in identifying proteins that may be low in abundance but crucial to the pathology under investigation, along with technical challenges that can result in inaccurate conclusions [51]. The FTIR spectroscopy approach, on the other hand, provides a spectrum that is representative of all analytes within the sample that have MIR active molecular bonds [52]. Therefore, the spectral “fingerprint” is not solely representative of the protein components of the samples but also lipids, sugars, and deoxyribonucleic acid (DNA) [34]. This is also the disadvantage of using FTIR spectroscopy, as it cannot be used to quantify compounds within a complex biological fluid (e.g., plasma) due to the overlap of the spectra of individual molecules within the sample. The unmixing approaches are available, but in the absence of pure compound controls, one is limited to blind unmixing heuristics, which may not necessarily guarantee robust, reproducible, or correct results. Therefore, FTIR spectroscopy should be considered as a complementary analytical approach to other quantitative instruments when the goal is the discovery of new biomarkers and/or putative therapeutic targets. The fact that FTIR spectroscopy performs better than MS in this study may be attributed to the influence of non-protein metabolites that are contributing to the observed differences (e.g., the metabolome). This can also be considered as an advantage.

A follow-up study is certainly warranted. Firstly, the FTIR spectra may be further validated in larger sample sets as a purely screening diagnostic biomarker without providing information on why the differences are observed. Secondly, the model performance based on the MIR spectra can be compared to other quantitative techniques (e.g., multi-omics) to determine what molecular components are contributing to the differences between the disease state and controls. The integration of information from various omics data types (i.e., genomics, epigenomics, transcriptomics, proteomics, lipidomics, and metabolomics) can provide a more global mapping of a disease state [20,21,53]. This global understanding of a disease state can aid in furthering knowledge of disease pathophysiology and provide targets for therapeutic interventions.

Conversely, the MS data enables the identification of proteins that account for the observed differences facilitating the interpretation of their roles in various cellular and molecular pathways. Following a multivariate feature selection process, certain proteins were determined to be particularly predictive. The overrepresentation analysis of these proteins indicated the activation of expected pathways, including increased platelet cytosolic Ca^2+^, platelet activation, signaling and aggregation, platelet degranulation, hemostasis, and neutrophil degranulation.

Interestingly, despite the strong individual performance of MS and FTIR spectroscopy in distinguishing FRI cases from controls, neither CCA analysis nor the cross-modal auto-encoder revealed a statistically significant link between the two modalities. Several (not mutually exclusive) mechanisms could explain this outcome. The first is simply power: with only 26 paired samples, high-dimensional noise can swamp any genuine cross-signal, whether linear or non-linear. A second possibility is biological complementarity: FTIR spectroscopy records a composite vibrational fingerprint of every chemical species in the specimen, whereas MS measures the relative abundance of specific proteins. Each platform may therefore be capturing different layers of the same pathological cascade, yielding complementary predictive cues. Conversely, the modalities might encode redundant biology in ways that remain uncorrelated because their feature distributions, dynamic ranges, or batch structures differ; latent confounders could drive parallel changes in both data sets without producing observable covariation.

Methodological limits also matter. CCA is restricted to linear projections, and our auto-encoder, constrained to a two-node bottleneck for over-fitting control, may be too small to capture complex, many-to-many mappings. Technical variance unique to each assay adds further unmodelled error. Taken together, these considerations make the current null result inconclusive rather than dispositive: the absence of detectable association does not imply that no mechanistic link exists. Resolving whether the spectroscopic fingerprint and the proteome intersect will require a much larger cohort and integrative models that can handle sparse, non-linear, and higher-dimensional relationships.

The effectiveness of both spectroscopy-based systems in achieving high predictability relies on performing feature selection. High dimensionality in spectral data can make classifiers significantly inefficient if entire, rich datasets are used without careful consideration. The feature selection process enhances classification performed with machine learning algorithms by eliminating irrelevant or redundant features from the dataset. This process is essential since including irrelevant features can adversely affect the accuracy and performance of the classification models [54]. By selecting the most relevant features, the dimensionality of the data is reduced, leading to several benefits, such as reducing measurement costs and storage requirements, coping with limited training sample sets, reducing training and utilization time, and facilitating data visualization and understanding [55]. Feature selection also helps to improve classification accuracy, reduce computation complexity, and enhance the performance of machine-learning models [56]. The feature selection process using the elastic net has been widely applied in various domains, including genetics, image processing, bioengineering, and other fields [57–59].

In terms of mechanistic explanations, the interplay between hemostasis pathways and infection, particularly in the context of sepsis and the immune response, is well established. The close interactions between immune defense and hemostasis have been extensively documented in bacterial infections, and hemostasis has been associated with an increased susceptibility to bacterial sepsis [60–62]. Activation of platelet degranulation-related pathways is an expected finding in areas of infection. Platelets possess the ability to internalize/entrap pathogens and confine them within engulfment vacuoles while also releasing platelet-derived cytokines that bolster the host’s defense against viral infections [63]. Moreover, their degranulation process not only directly eliminates pathogens but also modulates the activity of other immune cells, as platelets are capable of influencing the functions and recruitment of neutrophils, endothelium, and lymphocytes to sites of tissue damage or infection [64,65]. Another important pathway signifying the presence of an inflammatory response to the infection was neutrophil degranulation. Neutrophils serve as the initial line of defense for the body against invading pathogens, particularly bacteria [66]. When they are activated in a suitable manner, they release several pro-inflammatory cytokines and exhibit MHC Class II expression, which enables the presentation of antigen to T cells and triggers their activation [66].

Work-up of FRI is largely based upon history and physical exam, blood tests (i.e., white WBC, ESR, and CRP), radiographs, and occasionally advanced imaging (see Fig 1). However, WBC, ESR, and CRP have limited predictive value for FRI [13,14,67]. Quantitative histology and culture from intra-operative tissue samples are useful tools to diagnose FRI but require invasive testing and are dependent on sample quality. Further, quantitative histology is only validated for chronic or late-onset FRI [68], and culture results are not readily available intra-operatively, leading to delay in definitive treatment. Improved FRI diagnostic strategies are needed to turn clinical scenarios presenting with suggestive criteria into ones with confirmatory criteria [36] prior to the operating room. The data presented in this study are a step towards improved biomarkers for FRI diagnosis to overcome these limitations. Future studies to validate these methodology in larger population of patients can lead to adaptation of these tests into bed-side diagnostic tests to diagnose FRI non-invasively and follow patients during treatment to determine success would be valuable milestones [69].

The limitations of the study are the heterogeneous nature of fracture types included, small sample size, and single time-point sampling. However, despite these limitations, these preliminary results provide a baseline for further interrogation of MIR spectra-based FTIR spectroscopy and MS-based proteomic analysis in larger cohorts to continue the process of validating each technique as a diagnostic tool. If proven effective, the FTIR spectroscopy approach has several advantages over mass spectrometry, which can strengthen its use and transform it into a bedside test. These advantages include low cost, rapidity, simplicity, and adjuvant-free technique with minimal sample preparation requirements for conducting FTIR spectroscopy of biological fluids [34,35,39].

## Conclusion

The results of this study demonstrate that FTIR spectroscopy and MS-based proteomics are potential diagnostic biomarker candidates for FRI diagnosis. The predictive models based on the MIR spectra had better performance compared to MS protein abundance ratio data, which is likely due to the differences in methodologies and their measurement targets. The results of this study require further investigation and validation in larger cohorts of patients and additional time points of sample collection.

## Supporting information

S1 Table List of the proteins selected during the multivariate feature selection process. (DOCX)

S2 Table Pathways identified by Reactome knowledgebase for proteins in S1 Table. (DOCX)

S1 File Mass spectrometry methods. (DOCX)
