# Supplementary material for: Comparison of mass spectrometry and fourier transform infrared spectroscopy of plasma samples in identification of patients with fracture-related infections
Source: PLoS One. 2025 Sep 22;20(9):e0330743. doi: 10.1371/journal.pone.0330743 (PMC12453239; doi:10.1371/journal.pone.0330743)
Supplement: S1 Table — (DOCX) [file pone.0330743.s001.docx]

**S1 Table. List of the proteins selected during the multivariate feature selection process.**

| **Protein** | **Gene** | **Annotations** |
| --- | --- | --- |
| P02768 | ALB | Serum albumin; Serum albumin, the main protein of plasma, has a good binding capacity for water, Ca(2+), Na(+), K(+), fatty acids, hormones, bilirubin and drugs. Its main function is the regulation of the colloidal osmotic pressure of blood. Major zinc transporter in plasma, typically binds about 80% of all plasma zinc; Belongs to the ALB/AFP/VDB family |
| P06727 | APOA4 | Apolipoprotein A-IV; May have a role in chylomicrons and VLDL secretion and catabolism. Required for efficient activation of lipoprotein lipase by ApoC-II; potent activator of LCAT. Apoa-IV is a major component of HDL and chylomicrons; Belongs to the apolipoprotein A1/A4/E family |
| P03952 | KLKB1 | Plasma kallikrein; The enzyme cleaves Lys-Arg and Arg-Ser bonds. It activates, in a reciprocal reaction, factor XII after its binding to a negatively charged surface. It also releases bradykinin from HMW kininogen and may also play a role in the renin-angiotensin system by converting prorenin into renin; Belongs to the peptidase S1 family. Plasma kallikrein subfamily |
| P02766 | TTR | Transthyretin; Thyroid hormone-binding protein. Probably transports thyroxine from the bloodstream to the brain; Gla domain containing |
| P0C0L5 | C4B | Complement c4b (chido blood group); Complement C4-B; Non-enzymatic component of the C3 and C5 convertases and thus essential for the propagation of the classical complement pathway. Covalently binds to immunoglobulins and immune complexes and enhances the solubilization of immune aggregates and the clearance of IC through CR1 on erythrocytes. C4A isotype is responsible for effective binding to form amide bonds with immune aggregates or protein antigens, while C4B isotype catalyzes the transacylation of the thioester carbonyl group to form ester bonds with carbohydrate antigens |
| P04196 | HRG | Histidine-rich glycoprotein; Plasma glycoprotein that binds a number of ligands such as heme, heparin, heparan sulfate, thrombospondin, plasminogen, and divalent metal ions. Binds heparin and heparin/glycosaminoglycans in a zinc-dependent manner. Binds heparan sulfate on the surface of liver, lung, kidney and heart endothelial cells. Binds to N-sulfated polysaccharide chains on the surface of liver endothelial cells. Inhibits rosette formation. Acts as an adapter protein and is implicated in regulating many processes such as immune complex and pathogen clearance, cell chemotaxis, cell [...] |
| P01042 | KNG1 | Kininogen-1; (1) Kininogens are inhibitors of thiol proteases; (2) HMW-kininogen plays an important role in blood coagulation by helping to position optimally prekallikrein and factor XI next to factor XII; (3) HMW-kininogen inhibits the thrombin- and plasmin- induced aggregation of thrombocytes; (4) the active peptide bradykinin that is released from HMW-kininogen shows a variety of physiological effects: (4A) influence in smooth muscle contraction, (4B) induction of hypotension, (4C) natriuresis and diuresis, (4D) decrease in blood glucose level, (4E) it is a mediator of inflammation [...] |
| P81605 | DCD | Dermcidin; DCD-1 displays antimicrobial activity thereby limiting skin infection by potential pathogens in the first few hours after bacterial colonization. Highly effective against E.coli, E.faecalis, S.aureus and C.albicans. Optimal pH and salt concentration resemble the conditions in sweat. Also exhibits proteolytic activity, cleaving on the C-terminal side of Arg and, to a lesser extent, Lys residues |
| P05546 | SERPIND1 | Heparin cofactor 2; Thrombin inhibitor activated by the glycosaminoglycans, heparin or dermatan sulfate. In the presence of the latter, HC-II becomes the predominant thrombin inhibitor in place of antithrombin III (AT-III). Also inhibits chymotrypsin, but in a glycosaminoglycan-independent manner; Belongs to the serpin family |
| Q8NCM8 | DYNC2H1 | Cytoplasmic dynein 2 heavy chain 1; May function as a motor for intraflagellar retrograde transport. Functions in cilia biogenesis. May play a role in transport between endoplasmic reticulum and Golgi or organization of the Golgi in cells (By similarity); Dyneins, cytoplasmic |
| Q86YZ3 | HRNR | Hornerin; Component of the epidermal cornified cell envelopes; Belongs to the S100-fused protein family |
| Q9NSB4 | KRT82 | Keratin, type II cuticular Hb2; Keratins, type II |
| P29622 | SERPINA4 | Kallistatin; Inhibits human amidolytic and kininogenase activities of tissue kallikrein. Inhibition is achieved by formation of an equimolar, heat- and SDS-stable complex between the inhibitor and the enzyme, and generation of a small C-terminal fragment of the inhibitor due to cleavage at the reactive site by tissue kallikrein; Belongs to the serpin family |
| P01023 | A2M | Alpha-2-macroglobulin; Is able to inhibit all four classes of proteinases by a unique 'trapping' mechanism. This protein has a peptide stretch, called the 'bait region' which contains specific cleavage sites for different proteinases. When a proteinase cleaves the bait region, a conformational change is induced in the protein which traps the proteinase. The entrapped enzyme remains active against low molecular weight substrates (activity against high molecular weight substrates is greatly reduced). Following cleavage in the bait region, a thioester bond is hydrolyzed and mediates the c [...] |
| Q6UXB8 | PI16 | Peptidase inhibitor 16; May inhibit cardiomyocyte growth; CAP superfamily |
| O43866 | CD5L | CD5 antigen-like; Secreted protein that acts as a key regulator of lipid synthesis: mainly expressed by macrophages in lymphoid and inflammed tissues and regulates mechanisms in inflammatory responses, such as infection or atherosclerosis. Able to inhibit lipid droplet size in adipocytes. Following incorporation into mature adipocytes via CD36-mediated endocytosis, associates with cytosolic FASN, inhibiting fatty acid synthase activity and leading to lipolysis, the degradation of triacylglycerols into glycerol and free fatty acids (FFA). CD5L-induced lipolysis occurs with progression o [...] |
| P43652 | AFM | Afamin; Vitamin E binding protein. May transport vitamin E in body fluids under conditions where the lipoprotein system is not sufficient. May be involved in the regulation and transport of vitamin E at the blood-brain barrier; Belongs to the ALB/AFP/VDB family |
| Q96SB3 | PPP1R9B | Neurabin-2; Seems to act as a scaffold protein in multiple signaling pathways. Modulates excitatory synaptic transmission and dendritic spine morphology. Binds to actin filaments (F-actin) and shows cross-linking activity. Binds along the sides of the F-actin. May play an important role in linking the actin cytoskeleton to the plasma membrane at the synaptic junction. Believed to target protein phosphatase 1/PP1 to dendritic spines, which are rich in F-actin, and regulates its specificity toward ion channels and other substrates, such as AMPA-type and NMDA-type glutamate receptors. Pla [...] |
| P19823 | ITIH2 | Inter-alpha-trypsin inhibitor heavy chain H2; May act as a carrier of hyaluronan in serum or as a binding protein between hyaluronan and other matrix protein, including those on cell surfaces in tissues to regulate the localization, synthesis and degradation of hyaluronan which are essential to cells undergoing biological processes; Belongs to the ITIH family |
| P13645 | KRT10 | Keratin, type I cytoskeletal 10; Keratins, type I |
| O76096 | CST7 | Cystatin-F; Inhibits papain and cathepsin L but with affinities lower than other cystatins. May play a role in immune regulation through inhibition of a unique target in the hematopoietic system; Cystatins, type 2 |
| Q96PD5 | PGLYRP2 | N-acetylmuramoyl-L-alanine amidase; May play a scavenger role by digesting biologically active peptidoglycan (PGN) into biologically inactive fragments. Has no direct bacteriolytic activity; Belongs to the N-acetylmuramoyl-L-alanine amidase 2 family |
| P02751 | FN1 | Fibronectin 1; Fibronectin type III domain containing; Endogenous ligands |
| Q5SYB0 | FRMPD1 | FERM and PDZ domain-containing protein 1; Stabilizes membrane-bound GPSM1, and thereby promotes its interaction with GNAI1; FERM domain containing |
| P55103 | INHBC | Inhibin beta C chain; Inhibins and activins inhibit and activate, respectively, the secretion of follitropin by the pituitary gland. Inhibins/activins are involved in regulating a number of diverse functions such as hypothalamic and pituitary hormone secretion, gonadal hormone secretion, germ cell development and maturation, erythroid differentiation, insulin secretion, nerve cell survival, embryonic axial development or bone growth, depending on their subunit composition. Inhibins appear to oppose the functions of activins; Belongs to the TGF-beta family |
| Q9Y5Y7 | LYVE1 | Lymphatic vessel endothelial hyaluronic acid receptor 1; Ligand-specific transporter trafficking between intracellular organelles (TGN) and the plasma membrane. Plays a role in autocrine regulation of cell growth mediated by growth regulators containing cell surface retention sequence binding (CRS). May act as a hyaluronan (HA) transporter, either mediating its uptake for catabolism within lymphatic endothelial cells themselves, or its transport into the lumen of afferent lymphatic vessels for subsequent re-uptake and degradation in lymph nodes |
| P04278 | SHBG | Sex hormone-binding globulin; Functions as an androgen transport protein but may also be involved in receptor mediated processes. Each dimer binds one molecule of steroid. Specific for 5-alpha-dihydrotestosterone, testosterone, and 17-beta-estradiol. Regulates the plasma metabolic clearance rate of steroid hormones by controlling their plasma concentration |
| P02760 | AMBP | Alpha-1-microglobulin/bikunin precursor; Protein AMBP; Inter-alpha-trypsin inhibitor inhibits trypsin, plasmin, and lysosomal granulocytic elastase. Inhibits calcium oxalate crystallization; Lipocalins |
| P02765 | AHSG | Alpha-2-HS-glycoprotein; Promotes endocytosis, possesses opsonic properties and influences the mineral phase of bone. Shows affinity for calcium and barium ions; Cystatins, type 4 |
| Q6UY14 | ADAMTSL4 | ADAMTS-like protein 4; Positive regulation of apoptosis. May facilitate FBN1 microfibril biogenesis; ADAMTS like |
| Q8NCN4 | RNF169 | E3 ubiquitin-protein ligase RNF169; Probable E3 ubiquitin-protein ligase that acts as a negative regulator of double-strand breaks (DSBs) repair following DNA damage. Recruited to DSB repair sites by recognizing and binding ubiquitin catalyzed by RNF168 and competes with TP53BP1 and BRCA1 for association with RNF168-modified chromatin, thereby acting as a negative regulator of DSBs repair. E3 ubiquitin- protein ligase activity is not required for regulation of DSBs repair; Ring finger proteins |
| P30492 | HLA-B | HLA class I histocompatibility antigen, B-7 alpha chain; Involved in the presentation of foreign antigens to the immune system; C1-set domain containing |
| O14978 | ZNF263 | Zinc finger protein 263; Might play an important role in basic cellular processes as a transcriptional repressor; Belongs to the krueppel C2H2-type zinc-finger protein family |
| P02647 | APOA1 | Apolipoprotein A-I; Participates in the reverse transport of cholesterol from tissues to the liver for excretion by promoting cholesterol efflux from tissues and by acting as a cofactor for the lecithin cholesterol acyltransferase (LCAT). As part of the SPAP complex, activates spermatozoa motility; Apolipoproteins |
| Q15849 | SLC14A2 | Urea transporter 2; Specialized low-affinity vasopressin-regulated urea transporter. Mediates rapid transepithelial urea transport across the inner medullary collecting duct and plays a major role in the urinary concentrating mechanism; Solute carriers |
| P06396 | GSN | Gelsolin; Calcium-regulated, actin-modulating protein that binds to the plus (or barbed) ends of actin monomers or filaments, preventing monomer exchange (end-blocking or capping). It can promote the assembly of monomers into filaments (nucleation) as well as sever filaments already formed. Plays a role in ciliogenesis; Gelsolin/villins |
| P05452 | CLEC3B | Tetranectin; Tetranectin binds to plasminogen and to isolated kringle 4. May be involved in the packaging of molecules destined for exocytosis; C-type lectin domain containing |
| P15090 | FABP4 | Fatty acid-binding protein, adipocyte; Lipid transport protein in adipocytes. Binds both long chain fatty acids and retinoic acid. Delivers long-chain fatty acids and retinoic acid to their cognate receptors in the nucleus (By similarity); Belongs to the calycin superfamily. Fatty-acid binding protein (FABP) family |
| P00748 | F12 | Coagulation factor xii (hageman factor); Coagulation factor XII; Factor XII is a serum glycoprotein that participates in the initiation of blood coagulation, fibrinolysis, and the generation of bradykinin and angiotensin. Prekallikrein is cleaved by factor XII to form kallikrein, which then cleaves factor XII first to alpha-factor XIIa and then trypsin cleaves it to beta- factor XIIa. Alpha-factor XIIa activates factor XI to factor XIa |
