# Supplementary material for: Comparison of mass spectrometry and fourier transform infrared spectroscopy of plasma samples in identification of patients with fracture-related infections
Source: PLoS One. 2025 Sep 22;20(9):e0330743. doi: 10.1371/journal.pone.0330743 (PMC12453239; doi:10.1371/journal.pone.0330743)
Supplement: S2 Table — (DOCX) [file pone.0330743.s002.docx]

**S2 Table**. **Pathways identified by Reactome knowledgebase for proteins in Table S1**.

| **Pathway ID** | **Description** | **Identified proteins** |
| --- | --- | --- |
| R-HSA-114608 | Platelet degranulation | P01023, P01042, P02647, P02751, P02765, P02768, P04196, P05452, P29622 |
| R-HSA-76005 | Response to elevated platelet cytosolic Ca2+ | P01023, P01042, P02647, P02751, P02765, P02768, P04196, P05452, P29622 |
| R-HSA-140837 | Intrinsic Pathway of Fibrin Clot Formation | P00748, P01023, P01042, P03952, P05546 |
| R-HSA-8957275 | Post-translational protein phosphorylation | P01042, P02647, P02751, P02765, P02768, P05546, P19823 |
| R-HSA-381426 | Regulation of Insulin-like Growth Factor (IGF) transport and uptake by Insulin-like Growth Factor Binding Proteins (IGFBPs) | P01042, P02647, P02751, P02765, P02768, P05546, P19823 |
| R-HSA-76002 | Platelet activation, signaling and aggregation | P01023, P01042, P02647, P02751, P02765, P02768, P04196, P05452, P29622 |
| R-HSA-109582 | Hemostasis | P00748, P01023, P01042, P01871, P02647, P02751, P02765, P02768, P03952, P04196, P05452, P05546, P29622 |
| R-HSA-140877 | Formation of Fibrin Clot (Clotting Cascade) | P00748, P01023, P01042, P03952, P05546 |
| R-HSA-8963898 | Plasma lipoprotein assembly | P01023, P02647, P06727 |
| R-HSA-977225 | Amyloid fiber formation | P02647, P02766, P06396, P06727 |
| R-HSA-174824 | Plasma lipoprotein assembly, remodeling, and clearance | P01023, P02647, P02768, P06727 |
| R-HSA-8963899 | Plasma lipoprotein remodeling | P02647, P02768, P06727 |
| R-HSA-8963888 | Chylomicron assembly | P02647, P06727 |
| R-HSA-8963901 | Chylomicron remodeling | P02647, P06727 |
| R-HSA-8963896 | HDL assembly | P01023, P02647 |
| R-HSA-975634 | Retinoid metabolism and transport | P02647, P02766, P06727 |
| R-HSA-6806667 | Metabolism of fat-soluble vitamins | P02647, P02766, P06727 |
| R-HSA-8964058 | HDL remodeling | P02647, P02768 |
| R-HSA-2168880 | Scavenging of heme from plasma | P02647, P02760, P02768 |
| R-HSA-1474228 | Degradation of the extracellular matrix | P01023, P02751, P03952 |
| R-HSA-6798695 | Neutrophil degranulation | P02765, P02766, P06396, P30492, Q86YZ3 |
