# Supplementary material for: Comparison of mass spectrometry and fourier transform infrared spectroscopy of plasma samples in identification of patients with fracture-related infections
Source: PLoS One. 2025 Sep 22;20(9):e0330743. doi: 10.1371/journal.pone.0330743 (PMC12453239; doi:10.1371/journal.pone.0330743)
Supplement: S1 File — (DOCX) [file pone.0330743.s003.docx]

**S1 File. Mass spectrometry methods.**

*Mass Spectrometry Methods*

Mass spectrometry for this study was performed by the Center for Proteome Analysis (CPA) at Indiana University School of Medicine[1].

*Sample Preparation*

Plasma samples were allowed to thaw on ice and vortexed well. 10 µL of each were applied to room temperature mini-High-Select™ Top 14 abundant protein depletion spin columns (Thermo Fisher Scientific™ Cat No: A36370). As per vendor instructions, column and sample were incubated with end-over-end mixing for at least 10 minutes at room temperature. Proteins were recovered by centrifugation at 1,000 g for 2 minutes, then denatured and reduced by the addition of 1.5x volume 8 M urea in 100 mM 100 mM Tris pH 8.5, 10 mM tris(2-carboxyethyl) phosphine hydrochloride (TCEP, Sigma-Aldrich Cat No: C4706) for 30 minutes at room temperature. Proteins were then alkylated with a final concentration of 10 mM chloroacetamide (CAA, Sigma Aldrich Cat No: C0267) for 30 minutes at room temperature in the dark. Each sample was mixed with 0.4 µg Trypsin/LysC and incubated at room temperature for 3 hours prior to dilution with 50 mM Tris.HCl, pH 8.5 to a final urea concentration of 2 M, and overnight digestion at 35 ºC (Mass Spectrometry grade Trypsin/LysC, Promega Corporation, Cat No: V5072).

*Peptide Purification and Labeling*

Digestions were acidified with trifluoracetic acid (TFA, 0.5% v/v) and desalted on a Waters Sep-Pak 96-well plate, (Waters™, Cat no 186003966) with a wash of 1 mL 0.1% TFA followed by elution in 70% acetonitrile containing 0.1% formic acid (FA). Peptides were dried by speed vacuum and resuspended in 29 µL of 50 mM triethylammonium bicarbonate pH 8.0 (TEAB). Peptide quantitation was performed using Pierce Colorimetric Peptide Assay kit (Thermo Fisher Scientific™ Cat No: 23275).

*Tandem Mass Tag (TMT) Labeling and Fractionation*

Each sample was diluted with 50 mM TEAB pH 8.0 to a final concentration of 0.8 µg/µL and 25 µg peptides were labeled for two hours at room temperature with 0.2 mg of Tandem Mass Tag (TMTduplex™, Thermo Fisher Scientific, Cat No: 90063 Lot WG309126; Label 126 for control and 127 for infected plasma). Labelling reactions were quenched by adding 0.2% hydroxylamine (final v/v) to the reaction mixtures at room temperature for 15 minutes. Each set of control/infected labeled peptides were then combined, mixed, and dried by speed vacuum. After drying, samples were resuspended in 0.1% TFA and fractionated using a Waters Sep-Pak 96-well plate (Waters™, Cat no 186003966) with washes of 1 mL water followed by 1 mL 0.1% trimethylamine (TEA) 5 % acetonitrile, and elution fractions of 12.5%, 20%, and 70% acetonitrile containing 0.1% TEA. Fractions were dried using a speed vacuum and resuspended in 100 µL 0.1% FA.

*Liquid Chromatography-Mass Spectrometry (LC-MS)/MS Analysis*

Approximately 1/3 of each fraction (with three total fractions per matched set of digested plasma) was loaded onto Evotips (Evosep, Cat No: EV2001) as per manufacturer’s instructions, and analyzed on an Evosep LC with a 15 cm Endurance column (Evosep Cat No: 1106, 15 cm, ID150, 1.9µm) using the 15 sample per day (88min, EV1106) method. A fused silica emitter (Cat No: EV1087) was used with an EasySpray source (Thermo Fisher Scientific™) and FAIMSpro interface (Thermo Fisher scientific™) on an Exploris 480 orbitrap mass spectrometer (Thermo Fisher Scientific™). Chronos 2.0.11.0 was used to control the Evosep and trigger acquisition in Xcalibur (tune version 4.0.309.28). A positive ion mode, 5 FAIMS CV method was used with 0.6 sec cycle time per compensation voltage (-35, -45, -55, -65, -75 V CVs). APD was on with a default charge state of 2. For each cycle time, identical instrument parameters were used with MS1 settings of orbitrap resolution 60,000; scan range 400-1400 m/z, RF lens 40%; normalized AGC 200% (2e6) and max inject time of 50 ms. Monoisotopic peak determination was set to peptide with a minimum intensity of 2.5e4, charge states 2-7 and a shared dynamic exclusion of 60 s excluding isotopes and performing dependent scans on a single charge state per precursor. MS2 parameters were orbitrap resolution of 45,000, isolation window of 0.7 m/z, normalized collision energy of 35% HCD, fixed first mass of 100 m/z, normalized AGC target of 200% (2e5), and max IT of 105 ms.

**Reference**

1. Becker K, Sharma I, Slaven JE, Mosley AL, Doud EH, Malek S, et al. Proteomic Analyses of Plasma From Patients With Fracture-Related Infection Reveals Systemic Activation of the Complement and Coagulation Cascades. J Orthop Trauma. 2024;38(3):e111-e9. doi: 10.1097/bot.0000000000002752. PubMed PMID: 38117580; PubMed Central PMCID: PMCPMC10922838.
